# Using Biotechnology-Led Approaches to Uplift Cereal and Food Legume Yields in Dryland Environments

**DOI:** 10.3389/fpls.2018.01249

**Published:** 2018-08-27

**Authors:** Sangam L. Dwivedi, Kadambot H. M. Siddique, Muhammad Farooq, Philip K. Thornton, Rodomiro Ortiz

**Affiliations:** ^1^Independent Researcher, Hyderabad, India; ^2^The UWA Institute of Agriculture, The University of Western Australia, Perth, WA, Australia; ^3^Department of Crop Sciences, College of Agricultural and Marine Sciences, Sultan Qaboos University, Al Khoud, Oman; ^4^University of Agriculture, Faisalabad, Pakistan; ^5^CGIAR Research Program on Climate Change, Agriculture and Food Security (CCAFS), International Livestock Research Institute (ILRI), Nairobi, Kenya; ^6^Department of Plant Breeding, Swedish University of Agricultural Sciences, Alnarp, Sweden

**Keywords:** adoption and impact, applied genomics, gene editing, global warming, phenomics, photosynthesis, stress-tolerant cultivar, transgene

## Abstract

Drought and heat in dryland agriculture challenge the enhancement of crop productivity and threaten global food security. This review is centered on harnessing genetic variation through biotechnology-led approaches to select for increased productivity and stress tolerance that will enhance crop adaptation in dryland environments. Peer-reviewed literature, mostly from the last decade and involving experiments with at least two seasons’ data, form the basis of this review. It begins by highlighting the adverse impact of the increasing intensity and duration of drought and heat stress due to global warming on crop productivity and its impact on food and nutritional security in dryland environments. This is followed by (1) an overview of the physiological and molecular basis of plant adaptation to elevated CO_2_ (eCO_2_), drought, and heat stress; (2) the critical role of high-throughput phenotyping platforms to study phenomes and genomes to increase breeding efficiency; (3) opportunities to enhance stress tolerance and productivity in food crops (cereals and grain legumes) by deploying biotechnology-led approaches [pyramiding quantitative trait loci (QTL), genomic selection, marker-assisted recurrent selection, epigenetic variation, genome editing, and transgene) and inducing flowering independent of environmental clues to match the length of growing season; (4) opportunities to increase productivity in C_3_ crops by harnessing novel variations (genes and network) in crops’ (C_3_, C_4_) germplasm pools associated with increased photosynthesis; and (5) the adoption, impact, risk assessment, and enabling policy environments to scale up the adoption of seed-technology to enhance food and nutritional security. This synthesis of technological innovations and insights in seed-based technology offers crop genetic enhancers further opportunities to increase crop productivity in dryland environments.

## Introduction

Global warming overall is predicted to have negative effects on agricultural output and productivity. The current world population of 7.2 billion people is projected to increase to between 9.6 and 12.3 billion by 2100, mostly occurring in Africa and South Asia (Gerland et al., 2014). Current yield growth trends are insufficient to match the growing demand for food (Ray et al., 2013). Hence, meeting the dietary and nutritional requirements of an increasing population remains an enormous challenge.

Changes in weather pattern, erratic and uncertain rainfall, and rises in temperature have led to more frequent and longer dry spells and heat waves in many places. In addition to impacting crop growth and yields, such changes may affect crop performance by increasing infestations of insects and other pests, disease epidemics, and weed prevalence, and decreasing pollinating insects (Myers et al., 2017). Declining food quality and increased risk of food and feed contaminated with mycotoxin-producing fungi have the potential to adversely impact human and animal health (Dwivedi et al., 2013).

Agriculture accounts for 70% of freshwater use by humans, which is expected to increase by 17% by 2025 (Molden, 2007). Underground aquifers are rapidly being depleted due to excessive water use (Morison et al., 2008). The acreage of dryland agriculture will expand due to global warming (Challinor et al., 2014). Thus, drought and heat stress present critical challenges for enhancing crop productivity to ensure global food security (Lesk et al., 2016).

There are more than 160 million hectares of dryland cereal and legume crops worldwide. The regions where these crops are grown are prone to drought and heat stress, have limiting soil constraints, contain half of the global population, and account for 60% of the planet’s poor and malnourished people (Hyman et al., 2016). A recent study involving six major crop commodity groups in 131 countries reported losses from 1961–2014 of 1.6% in cereal production, 0.5% in oil crops, 0.6% in pulses, 0.2% in fruits, and 0.09% in vegetables due to drought and heat disasters. Estimated global production losses were US$ 237 billion (using 2004–2006 as a baseline), with the United States, the former Soviet Union, India, China, and Australia being the most adversely affected nations (Mehrabi and Ramankutty, 2017).

Three crop groups (cereals, legumes, and roots/tubers) are the major source of human food worldwide (1271.4 million tons), with root/tuber crops contributing to 65% (825 million t), cereals 26% (332 million t), and legumes 9% (114 million t) (http://www.fao.org/faostat/en/#data/QC, accessed on June 12, 2018). The major cereal crops are narley (*Hordeum vulgare* L.), maize (*Zea mays* L.), pearl millet (*Pennisetum glaucum* (L.) R.Br), rice (*Oryza sativa* L.), sorghum (*Sorghum bicolor* (L.) Moench) and wheat (*Triticum aestivum* L.). The major legume crops are chickpea (*Cicer arietinum* L.), common bean (*Phaseolus vulgaris* L.), cowpea (*Vigna unguiculata* (L.) Walp.), faba bean (*Vicia faba* L.), groundnut (*Arachis hypogaea* L.), lentil (*Lens culinaris* Medikus), peas (*Pisum sativum* L.), pigeonpea (*Cajanus cajan* (L.) Millsp.), and soybean (*Glycine max* (L.) Merr.). Groundnut and soybean are also the main sources of edible oil. Both cereals and legumes are covered in this review because they not only represent worldwide the most important crops, but both are also very similar in their responses to drought and heat stress (Nuñez Barrios et al., 2005; Prasad et al., 2008; Lopes et al., 2011; Daryanto et al., 2017).

In comparison to recent reviews with focus on understanding physiological and genetic basis of drought and heat stress tolerance (Kaushal et al., 2016; Dwivedi et al., 2017; Sita et al., 2017), assessing impact of drought stress on productivity of food crops at global level (Daryanto et al., 2017) and on germplasm mining using SNPs-based arrays and GWAS to identify SNPs/candidate genes associated with plant phenology, architecture, productivity, and stress tolerance (Dwivedi et al., 2017), this review has the unique focus on the use of genetic and genomic resources to enhancing stress [drought, heat, elevated CO_2_ (eCO2)] tolerance and productivity using biotech-led approaches in addition to the uptake and adoption of seed-based technology to enhancing food and nutritional security in dryland environments.

## Synthesis

### Physiological Basis of Adaptation to Stress Tolerance

#### Drought and Heat

Natural variation in response to drought or heat stress among and within crop species has been reported elsewhere (Dwivedi et al., 2010, 2013; Kaushal et al., 2016; Sita et al., 2017). The major physiological traits associated with drought or heat stress tolerance, both in cereals and legumes, include abscisic acid, canopy temperature, chlorophyll content, chlorophyll fluorescence (F_v_/F_m_), early vigor, harvest index (HI), membrane integrity, normalized difference vegetation index (NDVI), osmotic adjustment, photochemical efficiency, relative water content, restricted transpiration at high vapor pressure deficit (RT-HVPD) controlled by variation in hydraulic conductance and stomatal conductance, root architecture, stomatal conductance, stay-green (Stg), total transpiration (Tr), transpiration efficiency (TE), and water use efficiency (WUE; **Tables 1, 2**). Differences in the root profile, Stg, Tr, TE, HI, WUE, and RT-HVPD, among others, were associated with enhanced adaptation to drought and heat stress. Furthermore, the surrogate traits such as carbon isotope discrimination (δ^13^C), specific leaf area, and SPAD chlorophyll meter reading (SCMR) that determine WUE have been deployed in many breeding programs to enhance drought tolerance in both cereals and legumes (Rebetzke et al., 2002; Condon et al., 2004; Tausz-Posch et al., 2012; Cossani and Reynolds, 2015; Reynolds and Langridge, 2016).

**Table 1:** Physiological basis of drought and heat stress tolerance in grain legumes from 2005–2017.

Species	Physiological traits associated with stress tolerance	Reference
**Drought stress**		
Common bean (*Phaseolus vulgaris* L.)	Drought avoidance root traits (DART), water use efficiency (WUE), harvest index (HI), relative water content (RWC), stomatal conductance, pod harvest index	Beebe et al., 2013; Rosales et al., 2013; Polania et al., 2016; Medina et al., 2017
Chickpea (*Cicer arietinum* L.)	Canopy temperature, carbon isotope discrimination (δ^13^C), DART, early vigor, HI, low transpiration rate, restricted transpiration rate at high vapor pressure deficit (VPD), SCMR, transpiration efficiency (TE)	Kashiwagi et al., 2005, 2010, 2013; Zaman-Allah et al., 2011a,b; Krishnamurthy et al., 2012; Sivasakthi et al., 2017
Cowpea (*Vigna unguiculata* (L.) Walp)	Chlorophyll content, delayed leaf senescence, non-photochemical quenching, photosystem II, RWC, restricted transpiration rate at high VPD, TE	Kumar et al., 2008; Muchero et al., 2008; Belko et al., 2012; Mwale et al., 2017
Groundnut (*Arachis hypogaea* L.)	Chlorophyll content, HI, specific leaf area, SCMR, restricted transpiration rate at high VPD, TE, WUE	Upadhyaya, 2005; Devi et al., 2009, 2010; Devi and Sinclair, 2011; Hamidou et al., 2013; Vadez and Ratnakumar, 2016
Soybean (*Glycine max* L.)	Restricted transpiration rate at high VPD, slow wilting at high VPD associated with low leaf hydraulic conductance and early stomatal closure	Fletcher et al., 2007; Sinclair et al., 2008, 2010; Sadok and Sinclair, 2009; Seversike et al., 2013; Medina et al., 2017
**Heat stress**		
Chickpea	Chlorophyll content, membrane integrity, photochemical efficiency, pollen viability, RWC, seed setting, stigma receptivity, stomatal conductance	Kaushal et al., 2013
Cowpea	Membrane integrity, photosynthesis, pollen viability, stigma receptivity	Lucas et al., 2013; Angira, 2016
Groundnut	Efficient partitioning of photosynthate leading to high pod growth rate	Akbar et al., 2017
Soybean	Antioxidant metabolites (tocopherols, flavonoids, phenylpropanoids, and ascorbate precursors) confer tolerance to high temperature during seed development	Chebrolu et al., 2016

**Table 2:** Physiological basis of drought and heat stress tolerance in cereals from 2005–2017.

	Physiological traits associated with stress tolerance	Reference
**Drought stress**		
Barley (*Hordeum vulgare* L.)	Biomass, chlorophyll content, osmotic adjustment (OA), flag leaf photosynthesis rate, relative water content (RWC), stay-green, stomatal conductance	González et al., 2010; Vaezi et al., 2010; Gous et al., 2015; Sharma et al., 2016
Maize (*Zea mays* L.)	Restricted transpiration rate at high vapor pressure deficit (VPD) associated with low hydraulic conductance, root architecture, anthesis–silking interval (ASI), stay-green, water use efficiency (WUE)	Hund et al., 2009; Gholipoor et al., 2013a,b; Choudhary and Sinclair, 2014; Li et al., 2015; Messina et al., 2015; Maazou et al., 2016
Pearl millet (*Pennisetum glaucum* (L.) R.Br.)	Restricted transpiration rate at high VPD, tolerant and sensitive genotypes had similar total water extracted under drought stress; however, tolerant genotypes extracted less water prior to anthesis, and more water after anthesis	Kholová et al., 2010; Vadez et al., 2013; Medina et al., 2017
Rice (*Oryza sativa* L.)	Abscisic acid, carbon isotope discrimination (δ^13^C), chlorophyll content, membrane stability, photosynthesis rate, photosystem II activity, root architecture, RWC, stomatal conductance, transpiration rate, WUE	Kumar S. et al., 2014; Pandey and Shukla, 2015; Sandhu et al., 2016
Sorghum (*Sorghum bicolor* (L.) Moench)	Chlorophyll content, restricted transpiration rate at high VPD, stay-green, stomatal conductance	Xu et al., 2000; Gholipoor et al., 2010; Choudhary and Sinclair, 2014; Shekoofa et al., 2014
Wheat (*Triticum aestivum* L.)	Biomass, canopy temperature, chlorophyll content, δ^13^C, membrane integrity, HI, normalized difference vegetation index (NDVI), root architecture, small canopy, stay-green, stomatal conductance, water-soluble carbohydrate, WUE	Rizza et al., 2012; Nawaz et al., 2013; Wilson et al., 2015; del Pozo et al., 2016; Reynolds and Langridge, 2016; ElBasyoni et al., 2017; Nakhforoosh et al., 2017
**Heat stress**		
Barley	Chlorophyll content, stay-green	Gous et al., 2015
Maize	ASI, Canopy temperature, chlorophyll content, chlorophyll fluorescence(F_v_/F_m_), leaf firing, leaf senescence, membrane integrity, pollen shedding, seed setting, stigma receptivity, tassel blast, tassel sterility	Yadav et al., 2016; Alam et al., 2017
Pearl millet	Membrane integrity, percent seed setting	Yadav et al., 2014; Gupta et al., 2015
Rice	Anther and stigma characteristics, better antioxidant scavenging ability, membrane stability, photosynthesis, pollen development, RWC, seed setting	Jagadish et al., 2007, 2009; Bahuguna et al., 2014
Sorghum	Leaf firing, leaf blotching, floret fertility, individual seed weight, reduced seed setting	Prasad and Djanaguiraman, 2014; Prasad et al., 2015; Chen et al., 2017
Wheat	Canopy temperature, cell membrane stability, chlorophyll content, floret fertility, F_v_/F_m_, high early biomass, high grain filling rate, lower respiration rate, reduced seed weight, stay-green, stomatal conductance, transpiration	Prasad and Djanaguiraman, 2014; Kumari et al., 2013; Pandey et al., 2015; Sharma et al., 2015; Pinto et al., 2016, 2017; ElBasyoni et al., 2017

Drought and heat stress often occur together. Is tolerance to both genetically distinct from tolerance to either? Prasad et al. (2011) reported that heat reduced photosynthesis more than drought in wheat, while the lowest leaf photosynthesis observed was due to combined heat and drought stress. Each stress had similar effects on spikelet fertility, grain number, and yield (between 48 and 56%), while their combined effect was higher than their additive effects for chlorophyll content, grain number, and HI (Prasad et al., 2011). In maize, tolerance to combined drought and heat stress is due to distinct genes (Cairns et al., 2013). In chickpea, drought and heat stress individually damaged membranes and decreased cellular oxidizing ability, stomatal conductance, PSII function, and leaf chlorophyll content, with greater damage noted with the combined stress (Awasthi et al., 2014). Furthermore, a variable response to Rubisco (ribulose-1,5-biphosphate/oxygenase) activity was noted, which increased with heat, declined with drought, and declined significantly with both. Sucrose and starch concentrations declined significantly with combined stress. Drought stress had a greater effect on grain yield than heat stress, and the accessions showed partial cross-tolerance (Awasthi et al., 2014). In lentil, heat stress reduced seed yield more than drought stress, while the combined stress severely reduced seed size and seed weight, largely due to a reduction in sucrose and starch-synthesizing enzymes. The combined stress, however, had less effect on drought- and heat-tolerant lines, probably due to a partial cross-tolerance to the two stresses (Sehgal et al., 2017). Thus, the interactions between drought and heat stress should be considered when addressing these stresses in breeding programs.

#### Elevated CO_2_ (eCO_2_)

Atmospheric carbon dioxide (CO_2_) may increase from the current level (∼400 μmol mol^-1^) to 600 μmol mol^-1^ by 2050 (Ciais et al., 2013). eCO_2_ is associated with increased global warming (de Conto et al., 2012). Natural variation in grain yield in response to eCO_2_ has been noted in trials with cereals and grain legumes in controlled greenhouses or free-air CO_2_ enrichment (FACE) systems (**Table 3**), indicating the feasibility of exploiting genetic variation to develop cultivars responsive to eCO_2_. Reduced subsets (Frankel, 1984; Upadhyaya and Ortiz, 2001), representing the diversity of the entire collection of a given species in a genebank, are available for most cereal and grain legume crops (Dwivedi et al., 2006), and are excellent resources for identifying CO_2_-responsive germplasm.

**Table 3:** Cultivars response to elevated carbon dioxide (eCO_2_) relative to ambient carbon dioxide (aCO_2_) in barley, common bean, lentil, maize, pea, rice, soybean, and wheat from 2007–2017.

Germplasm no. (year/season)	Evaluation conditions	CO_2_ treatments	Summary of response at elevated CO_2_ (eCO_2_)	Reference
Barley (*Hordeum vulgare* L.)		
98 (2)	Open-top chambers	aCO_2_: 400 ppm; eCO_2_: 700 ppm	eCO_2_ increased aboveground biomass on average by 15% (range:36% to 95% among genotypes)	Mitterbauer et al., 2016
Common bean (*Phaseolus vulgaris* L.)		
4 (4)	Field open-top chambers	Ambient CO_2_ (aCO_2_): 370 μmol mol^-1^; eCO_2_: 550 μmol mol^-1^	Significant cultivar × CO_2_ interaction; seed yield at eCO_2_ was 0.89–1.39 times that of ambient CO_2_ (aCO_2_); pod/seed numbers at eCO_2_ the main determinant of response to seed yield	Bunce, 2008
Lentil (*Lens culinaris* Medik.)		
6 (3)	FACE	aCO_2_: 400 μmol mol^-1^; eCO_2_: 550 μmol mol^-1^	eCO_2_ increased yields by about 0.5 t ha^-1^, with greatest increase noted during terminal drought; relative increase among cultivars ranged from 18–138%; biomass increased by 32% and harvest index up to 60%	Bourgault et al., 2017
Maize (*Zea mays* L)		
3 (1)	Field open-top chambers	aCO_2_: 390 μmol mol^-1^; eCO_2_: 550 μmol mol^-1^	Biomass, grain yield and harvest index in relation to aCO_2_ improved by 32–47%, 46–27%, and 11–68%, respectively, under eCO_2_; grain number and test weight contributed to improved yield under eCO_2_	Vanaja et al., 2015
1(1)	Field open-top chambers	aCO_2_: ∼390 μmol mol^-1^; eCO_2_: ∼585 μmol mol^-1^	eCO_2_ had no effect on photosynthesis, biomass, or yield; reduced photosynthesis and a shift in aboveground carbon allocation contributed to reduced yield due to warming	Ruiz-Vera et al., 2015
1 (1)	Field SoyFACE facility	aCO_2_: 376 μmol mol^-1^; eCO_2_: 550 μmol mol^-1^	Photosynthesis and yield were not affected by eCO_2_ in the absence of drought	Leakey et al., 2006
Pea (*Pisum sativum* L.)		
5 (3)	FACE	aCO_2_: 390–400 ppm; eCO_2_: 550 ppm	eCO_2_ significantly increased seed yield by 26% due to increased pods per unit area, with no effect on grains per pod, grain size, or harvest	Bourgault et al., 2016
Rice (*Oryza sativa* L.)		
	FACE experiments	aCO_2_: 330–420 ppm; eCO_2_: 550–800 ppm	eCO_2_ increased yield by 20% despite no significant increase in grain size and weight; larger increase in belowground biomass than aboveground biomass; hybrid cultivars in relation to *japonica* or *indica* were more responsive to eCO_2_	Wang D.R. et al., 2016
4 (1)	Field open-top chambers	Varying CO_2_ and temperature (2°C > ambient temperature)	Grain yield increased with eCO_2_ and declined with elevated temperature across genotypes; however, some genotypes performed better than others	Dwivedi et al., 2015
3 cultivated and 1 wild rice (1)	Controlled environment chambers	Three temperature regimes (29/21, 31/23, 33/25°C)	eCO_2_ increased biomass and seed yield, however, response reduced due to increasing air temperature; weedy rice showed increased yield with eCO_2_ at all temperatures; ratio of tiller production between CO_2_ treatments at 30 DAS a significant predictor of seed yield in response to eCO_2_ at all temperatures	Ziska et al., 2014
24 (1)	Sunlit temperature-gradient chambers	aCO_2_: 377 μmol mol^-1^; eCO_2_: 568 μmol mol^-1^; temp: 23 and 18°C	eCO_2_ increased biomass by 27%, with tiller numbers the main driving force to increased biomass; significant genotype × temperature interaction; genotypes with higher biomass response to eCO_2_ had smaller reduction of biomass under low temperature	Shimono and Okada, 2013
4 (2)	FACE experiment; two planting density	aCO_2_: 383–385 μmol mol^-1^ eCO_2_: 576–600 μmol mol^-1^	eCO_2_ at low planting density increased biomass by 36–64%; significant cultivar × density interaction; panicle number and grain weight had similar response to eCO_2_; low planting density emulate eCO_2_ effects which could be used to select germplasm responsive to eCO_2_	Shimono, 2011
4 (2)	FACE experiments	aCO_2_: 383–385 μmol mol^-1^ eCO_2_: 576–600 μmol mol^-1^	eCO_2_ increased grain yield, with some cultivars more responsive than others; spikelet density contributed to increased response and enhancement in growth prior to heading could be a useful strategy to select cultivars responsive to eCO_2_	Shimono et al., 2009
16 (2)	Field open-top chambers	aCO_2_: 370 μmol mol^-1^ eCO_2_: 570 μmol mol^-1^	Genotypes responded differently to eCO_2_; yield increase ranged from 4–175% and 3–64% in two seasons; panicle number and grains per panicle contributed to greater yield at eCO_2_	de Costa et al., 2007
Soybean (*Glycine max* L.)		
12 (2)	Temp. gradient chambers in greenhouse	aCO_2_: 400 μmol mol^-1^; eCO_2_: 600 μmol mol^-1^; temp: 28°C	eCO_2_ increased seed yield up to 62% among cultivars, with aboveground biomass and pods plant^-1^ contributed most to the yield; low density (LD) increased yield by 5% to 105% owing to increased biomass and pods plant^-1^; yield increase in LD significantly associated with eCO_2_, suggesting biomass and pods plant^-1^ at LD could be used to select eCO_2_ responsive germplasm	Kumagai et al., 2015
18 (4)	FACE	aCO_2_: 380–390 ppm; eCO_2_: 550 ppm	Biomass and seed yield under eCO_2_ increased by 22% and 9%, respectively, but with reduced harvest index (HI); however, genotypes with highest HI under aCO_2_ also had highest HI in eCO_2_, suggesting HI could be used select for CO_2_ responsive germplasm	Bishop et al., 2015
Wheat (*Triticum aestivum* L.)		
4 (2) and 9 (1)	FACE	aCO_2_: 422 μmol mol^-1^; eCO_2_: 628 μmol mol^-1^	Significant differences among cultivars, evaluated on large and small plots in FACE, were detected, with some more responsive to eCO_2_; no loss in precision when response to eCO_2_ assessed on small plots	Bunce, 2017
2 (2); 3 growth stages	FACE	aCO_2_: 390 μmol mol^-1^; eCO_2_: 550 μmol mol^-1^	Drysdale (high transpiration efficiency, TE) responded more favorably to eCO_2_ than Hartog (low TE), with former yielding ∼19% more than Hartog; more green leaf mass (∼15%) and greater spike (∼8%) and tillers (∼11%) contributed to increased yield in Drysdale, suggesting cultivars with superior TE perform better under eCO_2_	Tausz-Posch et al., 2012
7 (1); 4 growth stages	Glasshouse	aCO_2_: 384 μmol mol^-1^; eCO_2_: 700 μmol mol^-1^	Grain yield increased by 38% under eCO_2_, correlated with spike number (*r* = 0.868) and aboveground biomass (*r* = 0.942); leaf mass per unit area (LMA) associated with increased N content on an area basis ([N]_LA_) and positively correlated with grain yield; differences in LMA could be used to select eCO_2_ responsive germplasm	Thilakarathne et al., 2012

aCO_2_, ambient carbon dioxide; eCO_2_, elevated carbon dioxide.

Establishing and operating CO_2_-fumigation facilities are costly. An alternative method is needed where large numbers of germplasm can be accommodated to assess genotype responses under eCO_2_. In multi-year and multi-location trials involving 127 diverse rice accessions, two planting densities (normal and wider spacing), Shimono et al. (2014) identified two *japonica* types most responsive to wider spacing as candidates to support the pre-screening CO_2_-responsiveness in a FACE system and temperature-gradient chambers under eCO_2_. The results from FACE confirmed that responsiveness to wider spacing density, as measured by changes in panicles plant^-1^, is positively associated with responsiveness to eCO_2_ across both temperate and tropical surroundings, suggesting that wider spacing density in rice would be a useful prescreen for testing diverse germplasm at low cost for responsiveness to eCO_2_ (Shimono et al., 2014). Finlay–Wilkinson’s regression coefficient (RC) approach has also been suggested as a preliminary evaluation to select accessions responsive to eCO_2_. In rice and soybean RCs and the responsiveness of yield to eCO_2_ were positively associated (Kumagai et al., 2016), suggesting that RCs may be initially used to discard accessions not responsive to eCO_2_. The reason for these relationships may be due to the high CO_2_-responsive accessions exhibiting high plasticity in resource-rich environments (Shimono, 2011; Shimono et al., 2014; Kumagai et al., 2015). Additional research is required to assess the validity of these methods in diverse crops.

Biomass, HI, heads per unit area, spikelet density, grains per panicle, and grain weight under eCO_2_ environments contributed to increased yield in cereals (Shimono et al., 2009; Shimono, 2011; Tausz-Posch et al., 2012; Thilakarathne et al., 2012; Shimono and Okada, 2013; Bunce, 2017), while increased biomass, HI, and pods and grains per plant enhanced yields in legumes (Bunce, 2008; Bishop et al., 2015; Kumagai et al., 2015; Bourgault et al., 2016, 2017). A detailed meta-analysis involving 79 species and eight reproductive traits showed that CO_2_ enrichment (500–800 μl l^-1^) across all species produced a greater proportion of flowers (19%), fruits (18%), and grains (16%), increased individual grain weight (4%) and test weight (25%), and lowered grain N (–14%). Crops and wild relatives showed a similar response for biomass; however, crops under eCO_2_ had higher HI and yielded more fruits (28% vs. 4%) and grains (21% vs. 4%) than the wild relatives. Thus, crops were more responsive to eCO_2_ than wild relatives (Jablonski et al., 2002).

Elevated CO_2_ is associated with increased atmospheric temperature. Both eCO_2_ and heat stress strongly affect crop production. There is a need to understand the genotypic response to such an interaction to identify genotypes responsive to eCO_2_ and heat stress. Rice and wheat grown under two levels of CO_2_ (ambient and enriched up to 500 μml mol^-1^) and temperature (ambient and increased 1.5–2.0°C) in FACE systems increased grain yield, while an increase in temperature reduced yield (Cai et al., 2016). These authors noted a differential response in the treatment combining eCO_2_ and temperature: greater reductions (17–35%) in wheat than rice (10–12%), and the number of filled grains per unit area accounted for most of the effect of eCO_2_ and temperature. Similar antagonistic effects of eCO_2_ and high temperature were noted among diverse rice accessions (Wang D.R. et al., 2016). Rising CO_2_ may compensate for losses due to drought in some situations. However, a recent study – conducted over eight years under varying precipitation and with year-to-year variation in weather at a unique open-air field facility – revealed that stimulation of soybean yield by eCO_2_ diminished to zero as drought intensified and eCO_2_ interacted with drought to modify stomatal function and canopy energy balance (Gray et al., 2016). eCO_2_ did not stimulate photosynthesis, biomass or grain yield, whereas the treatment combining eCO_2_ and high temperature reduced grain yield in maize (Ruiz-Vera et al., 2015). The above examples show the negative impact of rising CO_2_ on crop productivity due to changes in the intensity of drought and heat stress. Careful selection of crop cultivars responsive to eCO_2_ under drought and heat stress is needed, therefore, to minimize the negative impacts from interacting climatic factors in key crop production areas.

### Molecular Basis of Adaptation to Stress Tolerance

#### Drought

##### Legumes

A “quantitative trait loci (QTL) hotspot” region bearing 12 QTL for drought tolerance explained up to 58% of the variation in chickpea (Varshney et al., 2013). Further research involving this region unlocked two subregions enriched with 23 genes, of which four were functionally validated for drought tolerance in chickpea (Kale et al., 2015). Eighteen single nucleotide polymorphisms (SNPs) from five genes (*ERECTA, ASR, DREB, CAP2*, and *AMDH*) significantly contributed to enhanced drought and heat tolerance in chickpea (Thudi et al., 2014b). Roorkiwal et al. (2014) generated 1.3 Mbp of sequence data on 300 chickpea reference set accessions (Upadhyaya et al., 2008) using 10 genes known to confer drought adaptation, and noted 79 SNPs and 41 indels in nine genes, with the maximum number of SNPs in *ASR* gene, grouped into two distinct haplotypes.

In common bean, 19 *WRKY* genes, 11 downregulated and eight upregulated, responded to drought stress (Wu et al., 2017) and eight significant marker-trait associations under drought stress were noted on chromosome 9 and 11 (Hoyos-Villegas et al., 2017).

The stay-green QTL *Dro-1, Dro-3*, and *Dro-7* were noted in recombinant inbred lines and diverse germplasm, with a map resolution equal to or below ≤3.2 cM, suggesting valuable targets for marker-assisted genetic enhancement in cowpea (Muchero et al., 2013). Furthermore, the co-location of a stay-green trait with biomass and grain yield QTL (Muchero et al., 2009) opens up the opportunity to select for stay-green at the seedling stage as a rapid screening tool for selection of terminal drought tolerance in cowpea.

In pigeonpea (*C. cajan* (L.) Millsp.), U-Box protein-coding genes, *uspA* domain genes, *CHX* gene, and an uncharacterized protein gene (*C. cajan*08737) contribute to drought adaptation (Sinha et al., 2016).

Anderson et al. (2016) reported SNPs-based genetic variation associated with high temperature, low precipitation, or differing soil pH among wild soybeans. Accessions possessing related adaptation alleles could be deployed for soybean breeding. Environmental association analyses involving wild barley accessions unraveled QTL on chromosome 2H and 5H, which were related to temperature and precipitation, respectively (Fang et al., 2014). Multiple SNPs associated with drought and heat stress assessed by variations in total chlorophyll content, photochemical reflectance index, and δ^13^C among germplasm were found in soybean (Dwivedi et al., 2017). The *NAC* gene family enhances stress tolerance in plants. Eight of the 28 *GmNAC* genes are reportedly upregulated in drought-tolerant soybeans, which could be used in breeding to enhance abiotic stress adaptation (Hussain et al., 2017). *GmWRKY27* interacts with *GmMYB174* to enhance adaptation to drought stress in soybean (Wang F. et al., 2015). Wild soybean accessions contributed SNPs related to variability in reduced precipitation, heat stress, and soil pH (Anderson et al., 2016).

##### Cereals

In barley, 17 constitutively expressed (exclusively in tolerant accessions) and 18 drought-responsive (differential expression under drought among tolerant and intolerant accessions) genes have been reported (Guo et al., 2009). This suggests that both types contribute adaptation to drought stress in barley. A gene expression study involving barley accessions with varying levels of drought response unraveled 34 genes at the reproductive stage that were exclusively expressed in drought-tolerant accessions (Guo et al., 2009). *P5CS1* is the key gene involved in the biosynthesis of proline and is significantly induced under water-deficit conditions. Xia et al. (2017) detected 41 polymorphisms (16 SNPs and 25 indels) at the *HvP5CS1* locus among barley accessions, with 13 distinct haplotypes; of these, five polymorphisms in *HvP5CS1* were significantly (*P <* 0.001) associated with drought tolerance. Wild barley provided new genes and differentially expressed alleles for adaptation to drought. These genes appear to be more conserved than non-associated genes, while those tolerance genes evolved more rapidly than other drought-associated genes (Hübner et al., 2015).

Anthesis–silking interval (ASI), ears per plant, plant height, and stay-green traits were correlated with drought tolerance in maize (Messmer et al., 2009, 2011). Chromosome 3 bears a QTL hotspot region (∼8 Mb) harboring QTL for traits related to drought tolerance (Almeida et al., 2014). Many drought-tolerant candidate genes (271) and non-synonymous protein-coding SNPs (nsSNPs) (524), with most nsSNPs in bin 1.07 region harboring known drought-tolerant QTL, are reportedly involved in physiological and metabolic pathways in response to water deficit in maize (Xu et al., 2014). Li et al. (2016) detected numerous SNPs, located in 354 candidate genes, associated with drought-related traits. More recently, 77 SNPs associated with ten drought-responsive transcription factors involved in stomatal closure, root development, hormonal signaling, and photosynthesis were found in maize (Shikha et al., 2017). In a multi-environment study, Millet et al. (2016) noted a pattern of QTL effects expressed as functions of environmental variables, of which eight were associated with tolerance to heat and 12 with drought in maize.

A SNP related to a putative acetyl CoA carboxylase gene and an indel associated with a putative chlorophyll a/b binding protein gene, near a major drought-tolerant QTL, are linked with stay-green, grain yield, and HI in pearl millet under drought (Dwivedi et al., 2017).

In rice, several large-effect QTL have been reported in multiple genetic backgrounds and production environments (Kumar A. et al., 2014). In a study involving N22 (drought tolerant) and IR64 (drought intolerant) rice cultivars, Shankar et al. (2016) detected 801 transcripts that were exclusively expressed in N22 under stress conditions, with a larger number encoding NAC and DBP transcription factors. Transcripts encoding for thioredoxin and involved in phenylpropanoid metabolism were upregulated in N22. This suggests that cultivar-specific stress-responsive transcripts may serve as a useful resource to explore novel candidate genes for abiotic stress adaptation in rice. Plant phenotypic plasticity refers to producing a distinct phenotype in response to changing environments (Nicotra et al., 2010). Seventy-six SNPs and 233 priori candidate genes associated with root plasticity traits regulate root growth and development, which upon validation can be deployed in breeding to enhance rice adaptation to drought (Kadam et al., 2017). Mining allelic variability in the *OsDREB1F* gene in wild rice (*Oryza nivara* S. D. Sharma and Shastry) unlocked three SNPs conferring drought tolerance, which may be deployed to enhance drought stress adaptation in rice (Singh et al., 2015). Using image-based traits (i-traits) – non-destructive phenotyping as a measure of response to drought – Guo et al. (2018) noted high heritability and large variation and discovered previously unknown genes associated with drought resistance. For example, *OsPP15* is associated with a hub of i-traits, whose role was further confirmed by genetic transformation.

The reduction in canopy size associated with *Stg* QTL in sorghum reduces water demand prior to anthesis, thereby saving more water for plants during post-anthesis. Over expression of *P5CS2*, mapped within the *Stg1* QTL (Subudhi et al., 2000), in the stay-green line relative to the senescent line was correlated with higher proline (Johnson et al., 2015). Polymorphisms at *cis*-elements in the putative promoter region of *P5CS2* probably caused a difference in the expression of this gene, which may facilitate sorghum improvement by marker-assisted selection (MAS). Fracasso et al. (2017) assessed the gene expression dynamics of five drought-related genes in sorghum and found increased expression levels of all genes under drought in tolerant compared with sensitive genotypes, and identified three candidate genes (*SbCA, SbERECTA*, and *SbDHN*) that may be deployed to assess drought adaptation in sorghum. Five to nine SNPs near the 14 candidate genes conferred heat stress tolerance in sorghum (Chen et al., 2017).

Variation in water-soluble carbohydrates in stems confers stress tolerance in wheat (Blum, 1998). Polymorphisms of trait-associated SNPs unraveled eight candidate genes grouped into two distinct classes – defense response proteins and proteins triggered by environmental stress – which provide opportunities for selecting for stress tolerance in wheat (Dong et al., 2016). Meta-analysis unraveled 43 co-localized QTL for both drought and heat stress, and 20 drought and two heat stress QTL (Acuña-Galindo et al., 2015). Furthermore, integration of 137 SNP markers for drought- and heat-responsive candidate genes identified 50 candidate SNPs within MQTL confidence intervals, including genes involved in sugar metabolism, reactive oxygen species scavenging, and abscisic-acid-induced stomatal closure (Acuña-Galindo et al., 2015). More recently, three major QTL for drought-responsive traits, one each for days to anthesis (*QDa.ccsu-5A.1*), plant height (*QHt.ccsu-5A*), and 100-grain weight (*QTgw.ccsu-7A*), were reported under rainfed conditions among double haploid lines in wheat (Gahlaut et al., 2017). The maximum quantum efficiency of photosystem II, measured as *F_v_/F_m_*, is the most widely used parameter for the rapid non-destructive measurement of stress response in plants. Major QTL on chromosome 3B (two) and chromosome 1D (one), explaining 13–35% of the variation for *F_v_/F_m_*, involved in heat stress response were noted in wheat (Sharma et al., 2017).

Emmer wheat (*Triticum dicoccum* Schrank ex Schübl) – the durum wheat (*T. durum* Desf) ancestor – harbors a rich source of allelic diversity for abiotic stress adaptation (Peng et al., 2012). Near-isogenic lines (NILs) of durum and bread (*T. aestivum* L.) wheats containing wild emmer QTL alleles enhanced productivity and yield stability across environments, thereby enriching their gene pools with diversity for improving drought adaptation (Merchuk-Ovnat et al., 2016). Wheatgrass (*Agropyron elongatum* (Host) P. Beauv.) and wild barley (*Hordeum spontaneum* (K. Koch) Thell.) also provided genes for drought adaptation: *KNAT3* and *SERK1* in wheat (Placido et al., 2013) and *Hsdr4* in barley (*H. vulgare* L.) (Suprunova et al., 2007).

#### Heat

##### Legumes

Tolerance to heat stress is associated with browning (Hbs) of seed coats in cowpea. Five QTL associated with heat stress tolerance accounting for 11–18% phenotypic variation were tagged with 48 SNP in cowpea (Lucas et al., 2013). *Hbs-1* contributed 28–73% phenotypic variation associated with heat stress tolerance and segregated with SNPs, 1_0032 and 1_1128 (Pottorff et al., 2014).

##### Cereals

Genomic regions on chromosome 2H and 7H at elevated temperature and 17 root/shoot trait QTL have been found in barley (Dwivedi et al., 2017).

Six heat stress tolerant and 112 heat-responsive candidate genes were co-located within two QTL hotspot regions (Frey et al., 2015) – reported to contain three heat tolerance and 23 heat-responsive genes – for grain yield under heat stress in maize (Frey et al., 2016). Of interest is the heat tolerance gene *GRMZM2G324886*, present in both QTL hotspot regions, which encodes a calcicyclin-binding protein involved in calcium signaling in response to external stress (Frey et al., 2016).

Using NILs differing in heat sensitivity and cDNA-AFLP analysis, Liao et al. (2012) reported 54 transcript-derived fragments (TDFs), 45 of which were mapped to rice chromosomes, mostly in heat-tolerant lines. Twenty-eight of these homologous sequences encoding proteins were associated with signal transduction, oxidation, transcriptional regulation, transport, and metabolism; thus, they are good candidates for studying the molecular basis underlying adaptation to heat stress in rice. Five to 14 loci including *qHTSF4.1, qSTIPSS9.1*, and *qSTIY5.1/SSIY5.2*, which are major QTL associated with heat tolerance at the reproductive stage, are known in rice (Lafarge et al., 2017; Shanmugavadivel et al., 2017). A novel QTL on chromosome 9 for percent spikelet fertility at the reproductive stage and one known QTL on chromosome 5 for heat tolerance mapped to 331 kbp genomic regions, comprised 65 and 54 genes, respectively (Shanmugavadivel et al., 2017).

##### Elevated CO_2_ (eCO_2_)

The eCO_2_ in the atmosphere, which enhances CO_2_ fixation, plant growth, and production (Xu et al., 2013), is the major driving force behind global warming. The global surface temperature may rise between 2.6 and 4.8°C by the end of this century, depending on greenhouse gas emission pathway (IPCC, 2013). Stomata on the surface of plant leaves balance the uptake of CO_2_ with evaporation. How plants regulate their stomata in response to environmental stimuli may have important implications for future crop production. Stomata responds to eCO_2_ by partial closing, and large variation in response to eCO_2_ was noted among crop species [see section “Elevated Carbon Dioxide (eCO_2_)”]. The decrease in stomatal conductance, the rate at which CO_2_ enters the leaves, under eCO_2_ reduces water consumption and increases WUE. Stomatal function is regulated by genetic variation such as stomatal development, density, and trade-off, and molecular mechanisms regulating guard cell movement under eCO_2_, and its interactive effects with environmental factors such as vapor pressure deficit, temperature, CO_2_ concentrations, and light either alone or in combination (Xu et al., 2016). The genetic basis for inter- and intraspecific variation in the response of stomata under eCO_2_ and the mechanisms for sensing and responding to eCO_2_ are poorly understood in plants. To date, 16 genes (*HIC, GTL1, EPF2, STOMAGEN, OST1, CA1, CA4, SCAP1, HT1, APRC2, SLAC1, PATROL1, AtALMTA12/QAC1, QAC1, AtABCB14*, and *ROP2*), two (*EP3* and *SLAC1*) in rice, and one (*ROP2*) in faba bean, controlling stomatal development and movement response to eCO_2_ in *Arabidopsis*, have been discovered (Xu et al., 2016). Johansson et al. (2015) noted two genetic loci and candidate genes involved in the stomatal response to eCO_2_ in *Arabidopsis*. Furthermore, a MATE-type transporter associated with eCO_2_ concentration in the repression of *HT1*, which negatively regulates CO_2_-induced stomatal closing, was found in *Arabidopsis* (Tian et al., 2015). It was also shown that ABA in guard cells or their precursors mediate the CO_2_-induced stomatal density response in *Arabidopsis* (Chater et al., 2015).

### High-Throughput Phenotyping to Study Phenomes and Genomes and Their Use in Plant Breeding

High-throughput precision phenotyping (HPP) facilitates measurements within a wide spectrum of light reflectance wavelengths that should correlate significantly with a trait of interest to assess phenotypes (White et al., 2012; Yang et al., 2013). HPP and other phenomic methods and tools are becoming available to predict edible yield, nitrogen use efficiency, host plant resistance in early development stages, as well as for large-scale multi-environment trials (Furbank and Tester, 2011; Araus and Cairns, 2014). Phenomic platforms were first validated for their use in biotrons, growth chambers, and screenhouses, or for field testing using cameras attached to tractors and harvesters. Nonetheless, HPP remains limited in large-scale field trials because of the related costs and logistics involved in its implementation. The availability of relatively cheap unmanned aerial vehicles provides an opportunity for developing and adopting HPP to screen many genotypes evaluated in field trials. Adopting new technologies with efficient phenotyping may lead to larger genetic gains and reduce the time needed to breed climate-resilient cultivars. Phenomic tools such as digital imaging allow the screening of physiological traits that are difficult to measure in the field. Likewise, HPP platforms are facilitating functional genetics as noted in rice (Yang et al., 2013). Today selections are made that consider both the genotype and the resulting phenotype, which increases the speed and efficiency of selection.

Sir Ronald A. Fisher, Sewall G. Wright, and J. B. S. “Jack” Haldane defined quantitative genetic models that included various genes with large or small effects as well as non-genetic factors influencing the phenotype of complex traits, using variance components, resemblance between relatives, and the mathematical theory of selection, respectively (Ortiz, 2015). Large datasets of precise phenotypic measurements in early generation testing and large multi-environment trials are becoming available after using HPP methods to measure canopy temperature, or to obtain spectral reflectance indices and associated information, which can be used to predict grain yield and its components, as well as abiotic stress tolerance and host plant resistance to pathogens and pests.

Biometrics explains complex quantitative traits with continuous variation to understand the action of multi-genes and the genotype by environment interaction controlling phenotypic variation measured across environments. Furthermore, data from genome sequencing on genetic resources and breeding lines or populations along with HPP are increasing the knowledge base regarding specific genomic regions, loci, and alleles therein, haplotypes, linkage disequilibrium blocks, and gene networks contributing to specific phenotypes (Xu et al., 2012). This approach has changed plant breeding by enlarging it to a three-dimensional concept that includes genotype, phenotype and environment. Moreover, prediction models using high-density DNA markers offer promising results in terms of prediction accuracy (Abera Desta and Ortiz, 2014). Genomic prediction (GP) based on genotyping with genome-wide SNPs along with pedigree and phenotypic data is indeed powerful for capturing small genetic effects across the genome, thus allowing the prediction of breeding values or the genetic merit of an individual. Breeding values result from the parents’ average component and the Mendelian sampling of the parents’ genes. Genetic gains due to the response to selection depend on the accuracy and time taken to estimate the Mendelian sampling. The factors affecting the accuracy of GP are model performance, sample size and relatedness, DNA marker density, gene effects, trait heritability, and genetic architecture. Bayesian statistics can effectively estimate breeding values and may be coupled with geo-statistics for modeling multi-environment trials.

### Biotechnology-Led Methods to Enhance Crop Adaptation to eCO_2_, Drought, and Heat Stress

**FIGURE 1:**
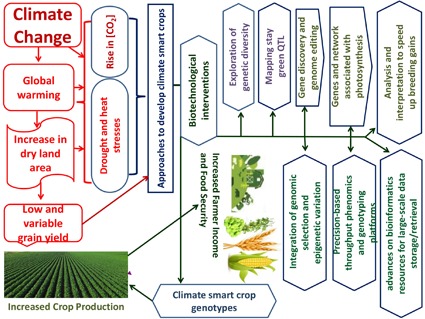
Biotechnological innovations to improve cereal and legume production in dryland environment under climate change.

Limited progress has been made on enhancing abiotic stress adaptation in crops through crossbreeding largely because of low heritability, genetic (epistasis) and genotype by environment interactions, and multigenic effects (Dwivedi et al., 2010). **Figure 1** shows how biotechnology may assist in developing cereal and legume-bred germplasm. Considerable progress has been made in developing genetic resources (i.e., diversity panels to identify new sources of variations and mapping populations to unravel the molecular basis of trait expressions) and genomic resources (i.e., high-density SNPs arrays, genetic maps, and reference genomes to unravel QTL and candidate genes associated with agronomically beneficial traits) for crop breeding. Transgenic research has moved from discovering protocols, promoters (constitutive or tissue-specific), and transgenes to developing appropriate technology to generate stress-tolerant GM crops without yield penalty under optimum agronomic conditions. Both genomic-assisted breeding and transgenic approaches can enhance the adaptation of cereal and legume crops to drought and heat stress, as detailed here.

### Pyramiding Quantitative Trait Loci

#### Drought Stress in Grain Legumes

A few chickpea introgression lines (ILs) harboring drought-tolerant QTL for root traits produced deeper roots, better root length density, and higher root dry weight than their recurrent parent J 11 or drought-tolerant donor parent ICC 4958 (Varshney et al., 2014), with some lines in national trials prior to release in India (Thudi et al., 2014a). Three backcross-derived cowpea (*V. unguiculata* L. Walp.) lines containing drought-tolerant QTL under water-deficit conditions produced on average 48% more seed yield (990–1185 kg ha^-1^) than the drought-tolerant cultivar Gorom (728 kg ha^-1^) in Burkina Faso (Batieno et al., 2016).

#### Drought Stress in Cereals

Six of 14 large-effect QTL associated with drought adaptation in rice were effective across genetic backgrounds and environments (Kumar S. et al., 2014). Pyramiding large-effect QTL enhanced drought adaptation of rice cultivars in Asia (Uga et al., 2013; Shamsudin et al., 2016; Dixit et al., 2017). Lines with two-*qDTY* combinations were more drought tolerant and productive than lines with three-*qDTY* combinations, suggesting a differential synergistic relationship among QTL (Shamsudin et al., 2016).

NILs containing *Stg* QTL in sorghum increased water uptake during grain filling relative to RT × 7000, and produced higher biomass, grain numbers, and grain yield without yield penalty across Australian environments (Borrell et al., 2014). In India, ILs containing *Stg* QTL in R16 (highly senescent but adapted to the post-rainy season) and ICSV 111 (dual purpose, grain and sweet sorghum types, open-pollinated cultivar) genetic backgrounds improved stover and grain yields as well as stover quality, with no tradeoff (Dryland Cereals and Archival Report, 2012–2013). A large-scale MABC program involving promising ILs and SSRs flanking *Stg* QTL was initiated to transfer stay-green trait into other sorghum cultivars adapted to the post-rainy season in India. Likewise, 1-2 *Stg* QTL was successfully transferred via marker-assisted breeding into a farmer-preferred sorghum cultivar E36-1 in Kenya (Ngugi et al., 2013).

The IL S42IL-121 containing major QTL from wild barley (*H. vulgare* ssp. *spontaneum*) produced 36% more biomass under drought-stressed conditions than recurrent parent Scarlet (Honsdorf et al., 2014). ILs containing major drought-tolerant QTL in pearl millet (*Pennisetum glaucum* (L.) R. Br) in the genetic background of a drought-sensitive parent (H 77/833-2) tolerated drought better and significantly out-yielded testcross hybrids made with the original recurrent parent under varying water deficits (Serraj et al., 2005).

#### Heat Stress in Cereals

*Oryza officinalis* contributed the major QTL *qEMF3* (on chromosome 3) for flower opening time in rice. NILs containing *qEMF3* in IR64 genetic background shifted flower opening time 1.5–2.0 h earlier in the tropics, thereby escaping heat stress (≥35°C causes spikelet sterility) at flowering. Thus, *qEMF3* has the potential to advance flower opening time in current cultivars to mitigate the effect of heat stress at flowering (Hirabayashi et al., 2015). *O. glaberrima* contributed a major QTL for heat tolerance *Thermotolerance1* (*TT1*), that protects cells from heat stress. NILs containing *TT1* allele had higher thermotolerance than the recurrent parent (WYJ) at flowering and grain filling (Li X. M. et al., 2015). Thus, pyramiding *qEMF3* and *TT1* is expected to enhance adaptation to heat stress in rice.

### Drought Tolerance in Marker-Aided Breeding in Maize

Ziyomo and Bernardo (2013) noted that indirect selection based on secondary traits or grain yield in maize under irrigation was not more efficient than selecting directly for grain yield under drought, while genomic selection (GS) was more efficient, with predicted relative efficiency of 1.24, for grain yield under water deficit. ASI, ear girth, ear length, 100-kernel weight, and grain yield in maize, on the other hand, had the highest prediction accuracies (0.95–0.97) across drought-stressed environments (Shikha et al., 2017). Furthermore, 77 SNPs distributed across the genomes were associated with ten drought-responsive transcription factors related to stomatal closure, root development, hormonal signaling, and photosynthesis (Shikha et al., 2017). A more recent study in maize revealed higher prediction accuracy of GS than phenotypic variance explained by the sum of QTL for individual traits following MAS. This suggests that using QTL-MAS in forward breeding will enrich allelic frequency for a few desired traits with strong additive QTL in early selection cycles while GS-MAS will additionally capture alleles with smaller additive effects (Cerrudo et al., 2018).

Marker-assisted recurrent selection (MARS) has been successfully employed to enhance genetic gains in maize, with greater genetic gains (105 kg ha^-1^ year^-1^) in well-watered (WW) than water-stressed (51 kg ha^-1^ year^-1^) plants in sub-Saharan Africa (Beyene et al., 2015). Likewise, test crosses derived from the second selection cycle (C_2_) had higher grain yield under drought stress than those coming from the original population, with a relative genetic gain of 7% per cycle, while test crosses of inbred S_1_ lines from C_2_ had an average genetic gain of 1% per cycle under WW and 3% per cycle under rainfed (Bankole et al., 2017). Hence, both GS and MARS were more effective at increasing genetic gain in maize under both irrigated and drought-stressed environments.

### Epigenetic Variation and Abiotic Stress Adaptation

Epigenetics is “*the study of mitotically and/or meiotically heritable changes in gene function that cannot be explained by changes in DNA sequences*” (Russo et al., 1996). Epigenetic changes contribute to gene expression under environmental stress and are regulated by processes such as DNA methylation, histone modification, and RNA interference (RNAi; Meyer, 2015). Increasing evidence shows that changes arising from DNA methylation or histone acetylation are heritable (Zheng et al., 2013; Wang W. et al., 2016) and may be exploited in plant breeding. Cytosine methylation polymorphism, as indicated by hypermethylation or hypomethylation under stress (Gayacharan and Joel, 2013), plays a key role in the expression of genes in plants (Shen et al., 2012). Hypermethylation and hypomethylation of DNA under drought are indicators of stress susceptibility and tolerance, respectively. The most commonly used assays to assess cytosine DNA methylation are methylation-sensitive amplified polymorphism (Xiong et al., 1999), bisulfite sequencing (Li et al., 2010), and methylated DNA immunoprecipitation sequencing (Taiwo et al., 2012).

#### DNA Methylation

DNA methylation is chemical modification arising from addition of a methyl group to the nitrogenous base in the DNA strand in a sequence-specific manner (Law and Jacobsen, 2010). Cytosine DNA methylation is a major epigenetic modification affecting gene regulation in response to environmental stress. Drought-induced genome-wide epigenetic modification accounted for ∼12% of the methylation differences in the rice genome, which were genotype-, tissue-, and developmental stage-specific (Wang et al., 2011), while Xia et al. (2016) noted greater DNA methylation (52.9–54.3%) under drought stress among diverse rice ecotypes adapted to upland (rainfed) and lowland (irrigated) sites. Moreover, adaptation-specific highly divergent epiloci confer adaptation to drought in both rice ecotypes (Xia et al., 2016). Gayacharan and Joel (2013) noted a positive correlation between spikelet sterility and methylation (%), whereas panicle length and weight, seeds per panicle, 100-seed weight, and grain yield were negatively correlated, suggesting the role of epigenetic regulation in grain-yield-attributing traits in response to drought in rice.

The molecular basis of DNA methylation at the genome level may unravel the regulatory mechanisms associated with abiotic stress adaptation. Wang W. et al. (2016) detected 1190 differentially methylated regions (DMRs) between drought-tolerant (IL_DK151) and intolerant (IR 64) lines under irrigation. The IL_DK151 plants under drought had more stable methylome (only 92 drought-induced DMRs) than IR64 plants (506 DMRs). Epigenetic regulation of drought responses was associated with changes in DNA methylation of genotype-specific genes. A set of 12 and 23 DMR-associated genes were differentially expressed in IL_DK151 and IR 64, respectively, under drought (Wang W. et al., 2016). Previous research found numerous DMRs among rice cultivars with distinct responses to drought and salinity, many of which were associated with the differential expression of genes associated with stress tolerance (Garg et al., 2015). Five of the six drought-stressed DMRs reported in faba bean (*V. faba* L.) had higher expression in drought tolerant (Bachar) than sensitive (F177) cultivars, suggesting their role in faba bean drought tolerance (Abid et al., 2017). DNA methylation polymorphism can, therefore, be effectively deployed to develop crops with enhanced drought tolerance.

#### Histone Modification

Histones are proteins associated with DNA in the nucleus that condense it into chromatin. Histone modifications may regulate gene expression in plants under abiotic stress. Post-translational modification of histones includes acetylation, methylation, phosphorylation, and ubiquitination (Chinnusamy and Zhu, 2009). Transcriptionally active chromatin is always associated with histone hyperacetylation, while inactive chromatin region is associated with deacetylated histone (Kim et al., 2008).

A change in the promoter region of cell cycle genes showing specific patterns of histone acetylation increased the total acetylation level under stress in maize roots. Differences in acetylation states (either hyper- or hypo-acetylation) of specific lysine sites on H3 and H4 tails of the promoter regions in cell cycle genes affect their expression under stress. Overexpression of *ZmDREB2A* enhances tolerance to osmotic stress in *Arabidopsis* (Qin et al., 2007). Furthermore, Zhao et al. (2014) noted that osmotic stress activates the transcription of the *ZmDREB2A* gene by increasing the levels of acetylated histones H3K9 and H4K5 associated with the *ZmDREB2A* promoter region.

Histone acetyltransferases (HATs) and histone deacetylases (HDACs) are involved in histone acetylation homeostasis. Eight HATs, grouped into four families, are known in rice. When investigating the response of four HATs, one from each family in rice, Fang et al. (2014) noted that drought significantly increased the expression of *OsHAC703, OsHAG703, OsHAF701*, and *OsHAM701*. They further showed that the acetylation level on certain lysine sites of H3 and H4 increased with *OsHATs* expression, suggesting that *OsHATs* is involved in the response of rice to drought. Previous research also identified that the modification in H3K4me3 was positively correlated with a subset of genes showing changes both in modification and expression under drought (Zong et al., 2013).

Programmed cell death (PCD) plays a prominent role in plants during development and in response to stress (Danon et al., 2000; Jones, 2001). Wang P. et al. (2015) showed significantly increased levels of total acetylation of histones H3K9, H4K5, and H3, whereas the di-methylation level of histone H3K4 remained unchanged and H3K9 decreased in maize seedlings under heat stress. Furthermore, maize seedlings treated with “trichostatin A” resulted in histone hyperacetylation caused by increased levels of the superoxide anion (O_2-_) within the cell, leading to PCD in association with histone modification changes under heat stress in maize leaves.

#### RNA Interference (RNAi)

MicroRNAs (miRNAs) are single-stranded RNA molecules (20–24 nt) that affect the expression of genes at the post-transcriptional level and are involved in plant development and a wide array of stress responses (Zhang et al., 2006). Many of the drought and heat stress-responsive miRNAs and their target genes associated with plant development, metabolism, and stress tolerance has been found in cereals and grain legumes. Drought-associated miRNAs include 11 in cowpea (Barrera-Figueroa et al., 2011), five in maize (Sheng et al., 2015), 18 each in rice (Barrera-Figueroa et al., 2012) and sorghum (Katiyar et al., 2015), and 71 in durum wheat (Liu et al., 2015). In contrast, the heat stress responsive miRNAs include eight in common bean (Jyothi et al., 2015), 11 and 26 in rice (Liu et al., 2017; Mangrauthia et al., 2017), and 6 and 12 in wheat (Xin et al., 2010; Kumar et al., 2015). Changes in miRNAs under stress correlate well with increased expression of stress-related genes in tolerant genotypes. For example, Liu et al. (2017) reported eight target genes that correspond with 26 miRNAs within the four QTL regions associated with heat stress in heat-tolerant rice. This relationship of miRNAs with their target genes, however, may not be one-to-one (i.e., negative or positive) and may vary between miRNAs of the same gene family or even for the same target miRNAs at various development stages (Lopez-Gomollon et al., 2012; Zhang et al., 2012; Kumar et al., 2015). Identification of differentially abundant miRNAs in stress-tolerant germplasm may thus provide molecular evidence that miRNAs contribute to stress tolerance and so are good candidates for enhancing stress tolerance in crops by transgenic breeding.

### Genome Editing to Harness Novel Allelic Variations in Stress Adaptation

Genome editing brings changes in DNA that are smaller than those from genetic engineering (Ainsworth, 2015). Rice and wheat showing host plant resistance to pathogens (Wang et al., 2014; Wang F. et al., 2016) or potato with improved cold storage and processing (Clasen et al., 2016) became available using genome editing.

Targeted modification of specific genes using CRISPR and CRISPR-associated proteinP9 (Cas9) leads to useful genetic variations for improving plants (Feng et al., 2013). Indeed, plant breeding enterprises are beginning to exploit genome editing with CRISP/Cas9 to develop genetically enhanced seed-embedded technology showing new traits. Avoiding off-target mutations during genome editing may be achieved using protocols to eliminate off-target effects and improve the specificity of the CRISPR/Cas-9 system (Pattanayak et al., 2013).

The *OPEN STOMATA2* (*OST2*) gene encodes the major plasma membrane H^+^-ATPase (AHA1) in *Arabidopsis* (Palmgren, 2001). Using CRISPR/Cas9 and the tissue-specific promoter *EF1* (*germline- and meristematic cell-specific*), Osakabe et al. (2016) generated a new mutant allele for *OST2/AHA1* (*ost2_crispr-1*) without off-target effects in *Arabidopsis*. Such plants did not show any significant differences in plant growth compared to wild-type (WT) plants. Furthermore, *ost2_crispr-1* plants exhibited higher leaf temperature and lower water loss than WTs, suggesting enhanced stomatal closure upon abiotic stress.

Maize plants overexpressing *ARGOS8* produced high grain yields under water deficit (Shi et al., 2015). Endogenous expression of *ARGOS8* mRNA in maize is low and spatially non-uniform. Using CRISPR/Cas9 technology and the GOS2 promoter, Shi et al. (2017) obtained heritable novel variants with enhanced expression of *ARGOS8* transcripts relative to the native allele in all maize tissues tested. Furthermore, such variants produced higher grain yield than their WT under water deficit at flowering, without any grain yield penalty under irrigation.

Rodríguez-Leal et al. (2017) recently proposed the engineering of beneficial quantitative variation for plant breeding through genome editing of promoters that leads to the generation of diverse *cis* regulatory alleles. They successfully generated tomato mutants from genome editing with variation in fruit size, inflorescence branching, and plant architecture. Their findings provide the underpinning for dissecting intricate interactions between gene regulatory changes and quantitative phenotypic variation, including adaptation to stress-prone environments.

### Inducing Flowering Independent of Environmental Clues to Match Growing Season

West Africa and South Asia are among the regions worst affected due to climate change and variability effects. Farmers in West Africa have experienced a shortening of the rainy season. Climate models have projected the late onset, early cessation of rainfall, and reduction in length of growing season, which will negatively impact agriculture in West Africa (Sarr, 2012) although there is high uncertainty about shifting rainfall patterns and amounts. In South Asia, by the end of the 21st century, models project a delay in the start of rainy season by 5–15 days and an overall weakening of the summer monsoon precipitation (Ashfaq et al., 2009). A reduction in the length of the growing season has direct impacts on the adaptation of current cultivars. There is thus a continued need to breed cultivars adapted to a shortened growing season. An alternative to crossbreeding is to engineer flowering time along with the external application of flower-inducing compounds (Ionescue et al., 2017).

Enhanced expression of the *Ghd7* gene under extended photoperiod delays flowering and increases plant height and panicle size in rice (Xue et al., 2008). Such orthologous floral repressor systems also exist in maize (*ZmCCT*) and sorghum (*SbPRR37* and *SbGHD7*) (Hung et al., 2012; Yang et al., 2014). Okada et al. (2017) produced non-flowering rice after overexpressing the *Ghd7* gene that inhibits environmentally induced spontaneous flowering. Thereafter, they isolated a promoter responsive to agrochemical spraying. Next, *Hd3a* was introduced under the control of an agrochemical-responsive promoter, which was already co-transformed with *Ghd7*. Such plants reacted to chemical spraying by beginning their floral transition, thus flowering earlier than the non-transgenic control, with no adverse impact on plant growth and development. This result shows that flowering time can be manipulated independently from the environment in which the plant grows, leading to the possibility of engineering crops with suitable growth in different climates (Okada et al., 2017).

Pyramiding QTL has been successful for combining tolerance to drought and heat stress into bred-germplasm for both cereals and legumes. Both GS and MARS, in contrast to QTL pyramiding by MAS, have been more effective at enhancing gains in maize productivity, both in drought-stressed and irrigated environments. The proof of concept also indicated the usefulness of deploying epigenetic variation or genomic editing to create new variation for agronomic traits and drought tolerance in some crops. As noted above, use of agrochemicals to induce flowering independent of environmental signals has also been effective, as evidenced in rice, thereby suggesting the utility of this approach in other crops.

### Stress-Tolerant Genetically Modified Crops

Plant adaptation to abiotic stress is achieved by genes associated with stress tolerance. The coordinated involvement of these genes and their interaction with the timing and intensity of the stress make it difficult for plant breeders to develop stress-tolerant crops by crossbreeding. Advances in genomics are allowing researchers to identify tissue-specific expression of genes under abiotic stress. The transfer and expression of such candidate genes into crops through genetic engineering offer considerable promise.

The overexpression of transgenes enhances abiotic stress adaptation, both in model plants and cereals (Dwivedi et al., 2010). Grain yield penalty is often associated with the introduction of transgenes that improve plant responses to abiotic stress adaptation (Campos et al., 2004). Overexpression of *HvEPF1* in barley, *TPP* in maize, *OsERF48, OsKAT2, AtGolS2, OsERF71, OsNAC9*, and *OsNAC10* in rice, *AtGoLS2* in soybean, and *SeCspA* and *TaNAC69-1* in wheat enhances drought adaptation and increases grain yield under field drought, without yield penalty under WW conditions (**Table 4**). Overexpression of *OsMYB55* enhances adaptation to drought and heat stress in maize and rice (El-Kereamy et al., 2012; Casaretto et al., 2016). Furthermore, *OsMYB55* under individual or combined (drought and heat) stress during or after recovery of stress produced more plant biomass with less leaf damage in maize (Casaretto et al., 2016). It appears that using a tissue-specific promoter (i.e., root-specific or seed-specific), rather than whole body (plant)-specific promoters, containing a transgene had no or relatively fewer adverse effects on plant growth and development, leading to no yield penalty.

**Table 4:** Overexpression of transgenes enhances drought adaptation and increases grain yields under field-drought environments in barley, maize, rice, soybean, and wheat from 2010–2018.

Species	Gene	Effect of transgene	Reference
Barley (*Hordeum vulgare* L.)	*HvEPF1*	Reduced stomatal density and enhanced water use efficiency, drought tolerance, and soil water conservation properties with no yield penalty	Hughes et al., 2017
Maize (*Zea mays* L.)	*OsMYB55*	Increased plant biomass and reduced leaf damage under drought and heat stress (individual or combined) during or after recovery from stress	Casaretto et al., 2016
	*TPP*	Enhanced grain yield by 9–49% under moderate or 33–123% under severe drought conditions	Nuccio et al., 2015
Rice (*Oryza sativa* L.)	*ZmPIF1*	*ZmPIF1*, a positive regulator of ABA signaling pathway, confer drought tolerance by regulating stomatal aperture; tiller and panicle numbers associated with increased grain yield	Gao et al., 2018
	*OsERF48*	Root-specific (ROX*^OsERF48^*) or whole-body specific (OX*^OsERF48^*) overexpression resulted longer and denser roots compared to wild-type; ROX*^OsERF48^* plants exhibited higher grain yield than OX*^OsERF48^* or wild-type plants under field-drought conditions	Jung et al., 2017
	*OsKAT2*	Delayed photo-induced stomatal opening, reduced water loss, and increased drought tolerance, with no yield penalty	Moon et al., 2017
	*AtGolS2*	Conferred drought tolerance and increased grain yield under drought-stressed conditions in two genetic backgrounds	Selvaraj et al., 2017
	*OsERF71*	Enhanced drought tolerance at reproductive stage and increased grain yield by 23–42% over wild-type	Lee et al., 2016
	*SNAC3*	Enhanced tolerance to drought and high temperature	Fang et al., 2015
	*HYR*	Improved photosynthesis leading to increased grain yield under drought and heat stress conditions	Ambavaram et al., 2014
	*OsNAC9*	Enhanced drought tolerance and increased grain yield by 28–72% under field-drought conditions	Redillas et al., 2012
	*OsMYB55*	Improved tolerance to high temperature and increased plant biomass	El-Kereamy et al., 2012
	OsNAC10	Improved drought tolerance and increased grain yield by 25–42% and by 5–14% over controls under drought and irrigated conditions, respectively	Jeong et al., 2010
Soybean (*Glycine max* L.)	*AtGoLS2*	Enhanced drought tolerance and improved seed yield under field-irrigated and drought-stressed environments	Honna et al., 2016
Wheat (*Triticum aestivum* L.)	*TaFER-5B*	Exhibited enhanced drought and thermotolerance	Zang et al., 2017
	*SeCspA*	Enhanced drought tolerance and increased seed weight (19–20%) and seed yield (24%)	Yu et al., 2017
	*TaNAC69-1*	Enhanced primary root length and root biomass, produced 32 and 35% more aboveground biomass and grains, respectively, under water-deficit conditions	Chen et al., 2016

Selection criteria in breeding programs will benefit from insights on the molecular and physiological basis for adaptation to increased grain yield under stress. For example, *HvEPF1* significantly reduced stomatal density but enhanced WUE and soil water conservation properties, thus enhancing drought adaptation and productivity in barley (Hughes et al., 2017). Likewise, improving sucrose supply or altering sucrose metabolism in developing ear spikelets improved kernel set in maize under drought (Zinselmeier et al., 1995; Boyer and Westgate, 2004). Furthermore, *EPP* increased sucrose content in spikelet and kernel set in maize, leading to increased HI and grain yield under drought, without yield penalty under WW conditions (Nuccio et al., 2015). Similarly, in rice, *OKAT2* delayed photo-induced stomatal opening and enhanced drought adaptation without grain yield penalty (Moon et al., 2017), while tolerance to heat stress seems to be related to enhanced amino acid metabolism by *OsMYB55* (El-Kereamy et al., 2012).

Environmental stress reduces photosynthetic carbon metabolism (PCM) and limits grain yield in cereals. For example, increased expression of *HYR*, which is associated with PCM, enhanced photosynthesis, leading to high grain yield in rice under stress (Ambavaram et al., 2014). Overexpression of *OsERF48, OsERF71, OsNAC9*, and *OsNAC10* enhanced grain yield under water scarcity by altering root architecture (Jeong et al., 2010; Redillas et al., 2012; Lee et al., 2016; Jung et al., 2017). In contrast, increased grain yield in transgenic rice overexpressing *AtGolS2* under drought was related to higher panicle numbers, grain fertility, and biomass (Selvaraj et al., 2017). Enhanced thermotolerance due to overexpression of *TaFER-5B* in wheat was due to ROS scavenging (Zang et al., 2017). Overexpression of *TaNAC69-1* under drought promoted root elongation in drying soils, producing more aboveground biomass and grain in wheat (Chen et al., 2016), while overexpression of *SeCspA* under drought increased test weight and grain yield (Yu et al., 2017).

Monsanto’s “DroughtGuard” maize (MON87460) expressing *Bacillus subtilis* cold shock protein B (cspB) (Castiglioni et al., 2008) which produced 6% higher grain yield across years than the control (Monsanto hybrid NH6212) under water-limited conditions (Nemali et al., 2015) was cultivated on ≥50,000 ha in the United States during the first year after release (Marshall, 2014). New genes conferring drought and heat stress adaptation are being discovered and the experimental area sown to drought or heat stress tolerant crops containing transgene(s) is expected to grow as more countries become involved in the development and field testing of such crops. However, the major concern with biotech crops is food safety for humans and animals. A rigorous assessment based on robust, science-based criteria should be in place to assess biosafety issues associated with the use of such crops.

Unlike in the past when the induction of transgenes was often associated with adverse impacts on plant growth and productivity, the proof of concept has been demonstrated for the development of stress tolerant genetically modified crops without yield penalty under agronomically optimal environments, as evidenced both in cereals and legumes.

### Genetic Variation and Candidate Genes Associated With Photosynthetic Traits

Photosynthesis is a complex process whereby green plants use sunlight to synthesize nutrients (carbohydrates) from carbon dioxide (CO_2_) and water, in the process, generating oxygen as a by-product. The process of converting light energy into chemical energy to produce biomass is highly efficient. Over the years, there has been increased understanding of the processes that limit photosynthetic efficiency (PE), paving the way to manipulate light energy into carbon gain in plants. Future increases in biomass production may, therefore, depend largely on increases in PE, with a focus on harnessing cross species (C_3_ vs. C_4_ crops) as well within-species differences, as detailed below.

#### Harnessing Novel Variation for Photosynthesis Traits

Plant genetic resources are a valuable foundation of natural genetic variation for photosynthetic traits. Rubisco is a target for improving photosynthesis. Prins et al. (2016) measured Rubisco carboxylation velocity (V_c_), Michaelis–Menten constants for CO_2_ (K_c_) and O_2_ (K_o_), and specificity factor (S_c/o_) for Rubisco diversity *in vitro* in 25 accessions of the *Triticeae* tribe at 25 and 35°C and noted a positive association between V_c_ and K_c_ and negative association between V_c_ and S_c/o_. Rubisco from barley increased photosynthesis at 25 and 35°C while Rubisco from jointed goatgrass (*Aegilops cylindrica* Host) increased photosynthesis at 25°C. Thus, naturally occurring Rubisco with superior properties among the *Triticeae* tribe may be exploited to develop wheats with enhanced photosynthesis and productivity. The CO_2_ fixation rate (K_ccat_) for Rubisco from the C_4_ grasses (*Paniceae* tribe) with NAD, NADP-ME, and PCK photosynthetic pathways was twofold greater than the *k*ccat of Rubisco from NAD-ME species across all temperatures, while the declining response of CO_2_/O_2_ specificity with increasing temperature was less pronounced for PCK and NADP-ME Rubisco, which would be advantageous in warm climates relative to the NAD-ME grasses (Sharwood et al., 2016). Rubisco from certain C_4_ species could thus enhance carbon gain and resource use efficiency in C_3_ crops.

Driever et al. (2014) reported significant variation in photosynthetic capacity, biomass, and grain yield among 64 wheat accessions, although they noted an inconsistent correlation between the flag leaf photosynthetic capacity and grain yield across accessions, thus suggesting scope for further improvement in photosynthetic capacity. Moreover, Carmo-Silva et al. (2017) compared flag leaf photosynthetic traits, crop development, and grain yield in the same cultivars used by Driever et al. (2014) and noted that pre-anthesis and post-anthesis photosynthetic traits in the field were positively associated with grain yield and HI. Moderate to high heritability for photosynthetic traits suggests that phenotypic variation can be deployed to enhance photosynthesis in wheat.

Gu et al. (2012a) noted significant genetic variation for stomatal conductance (G_s_), mesophyll conductance (G_m_), electron transport capacity (J_max_), and Rubisco carboxylation capacity (V_cmax_) in rice, although drought and leaf age contributed for a greater proportion of phenotypic variation. The differences in light-saturated photosynthesis and TE were mainly associated with variation in G_s_ and G_m_. Further research revealed that a known photosynthesis-QTL is associated with differences in Gs, G_m_, J_max_, and V_cmax_ at flowering. One to three QTL associated with eight photosynthetic traits contributed 7–30% of the phenotypic variation, with some QTL near the SSR marker RM410 consistent across development stages and moisture regimes (Gu et al., 2012b). Gu et al. (2014) noted a 25% increase in genetic variation in leaf photosynthetic rate (P_r_) corresponding to a 22–29% increase in crop biomass across locations and years. More recently, Qu et al. (2017) detected large variation in photosynthetic parameters among diverse rice germplasm across environments, and noted that P_r_ under low light intensity is correlated with biomass accumulation, and the narrow-sense heritability is high, suggesting its potential in breeding for improving biomass and yield potential in rice.

Natural variation in the CO_2_ assimilation rate is another promising strategy to identify genes contributing to higher photosynthesis. Adachi et al. (2017) fine mapped a QTL *Carbon Assimilation Rate8* (*CAR8*), identical to rice flowering QTL *DTH/Ghd8/LHD1*, that controls flag leaf nitrogen content, G_s_ and P_r_ in rice. QTL *GPS* (*GREEN FOR PHOTOSYNTHESIS*), identical to *NAL1* (a gene that controls lateral leaf growth in rice), controls photosynthesis rate by regulating carboxylation efficiency. The high-photosynthesis allele of *GPS* is a partial loss-of-function allele of *NAL1*, which increases mesophyll cells between vascular bundles, leading to thickened leaves, and pleiotropically enhances P_r_ with no adverse impact on plant productivity (Takai et al., 2013). Further research on molecular functions of *GPS* and *CAR8* may contribute to enhanced photosynthesis in rice and possibly other crops.

Understanding the genetic basis of PE may contribute to enhanced seed yield. Basu et al. (2018) reported 16 SNPs linked to major QTLs underlying 16 candidate genes associated with PE and seed yield in chickpea. Furthermore, they delineated superior haplotypes from a chlorophyll A-B binding protein-coding gene, *Timing of a CAB Expression1*, as the most potential candidates for enhancing PE and seed yield.

Enhancing G_m_ may increase WUE and photosynthetic water use efficiency (WUE_p_). G_m_ refers to the rate of CO_2_ diffusion from substomatal cavities to the site of carboxylation. To improve WUE, G_m_ should increase without concomitantly increasing G_s_. Across species, a 24-fold variation was noted in G_m_ (Tomás et al., 2013). Within-species differences in G_m_ were also noticed, e.g., 60% in rice (Gu et al., 2012a), two to threefold in wheat (Jahan et al., 2014; Barbour et al., 2016), and twofold in soybean (Tomeo and Rosenthal, 2017). A QTL for G_m_ on chromosome 3A contributes to 9% variation in wheat (Barbour et al., 2016). Tomeo and Rosenthal (2017) found a positive correlation between G_m_ and P_r_ in soybean; however, it impedes positive scaling between G_m_ and WUE. Both P_r_ and G_m_ increased with increasing leaf mass, suggesting the potential to increase photosynthesis and G_m_ by selecting for greater leaf mass in soybean (Tomeo and Rosenthal, 2017).

WUE_p_ refers to carbon fixed in photosynthesis per unit of water transpired (Lawson and Blatt, 2014). ILs with a high density of hairs on leaves containing a hairy leaf gene *BLANKET LEAF* from *O. nivara* in IR24 had a warmer leaf surface, and lower net photosynthesis rate (P_n_), transpiration rate (T_r_), and G_s_, but higher WUE_p_, suggesting that *BLANKET LEAF* increases WUE_p_ when evaluated under moderate to high light intensities. Hence, it could be deployed to improve WUE_P_ in rice (Hamaoka et al., 2017). ILs containing a genomic region from *O. rufigopon* in KMR3 showed significant variation in P_n_, T_r_, TE (P_n_/T_r_), and carboxylation efficiency (P_n_/C_i_). Hamaoka et al. (2017) further noted positive correlations involving P_n_ with T_r_, G_s_, P_n_/C_i_, and total canopy dry matter, and several ILs had higher *p_n_*. Thus, ILs with greater P_n_ are potential sources for breeding rice cultivars with enhanced biomass and yield (Haritha et al., 2017).

#### Transforming C_3_ Plants With C_4_ Photosynthesis

C_4_ plants depending on the mechanisms of decarboxylation of C_4_ acids in bundle sheath cells are grouped as NADP-malic enzyme (NADP-ME), NAD-malic enzyme (NAD-ME), and PEP carboxykinase (PCK) biochemical types (Hatch, 1987). Genus *Flaveria* consists of species that perform C_3_, C_4_, and C_3_–C_4_ (intermediate) photosynthesis. All *Flaveria* species are closely related and therefore may unravel key mutations in the evolution from C_3_ to C_4_ photosynthesis (Monson et al., 1986; Brown et al., 2005). Fixation of CO_2_ in C_3_-plants is catalyzed by the enzyme Rubisco. However, Rubisco is prone to energy loss due to photorespiration under high temperatures. In contrast, C_4_ plants have developed an additional CO_2_ concentration mechanism to minimize this energy loss, which enables them to adapt to abiotic stresses (Peterhänsel and Maurino, 2011; Sage et al., 2012). The key enzyme in the C_4_ photosynthesis pathway is phosphoenolpyruvate carboxylase that, during the evolution of C_4_ photosynthesis, increased its kinetic efficiency and reduced its sensitivity to feedback inhibitors (malate, aspartate). A single point mutation from “arginine” at the inhibitor site (884) to “glycine” in the active site (774) reduces inhibitor affinity and enables phosphoenolpyruvate carboxylases to participate in the C_4_ photosynthesis pathway (Paulus et al., 2013).

Amaranth (*Amaranthus palmeri* S.Wats.) is the first dicotyledonous NAD-ME-type C_4_ species possessing “Kranz anatomy” (i.e., tightly arranged, thick-walled, vascular bundle sheath cells, and surrounding mesophyll cells with airspaces) with P_n_ similar to C_4_ species. It is highly resource-use efficient and tolerant to drought, heat, and salinity (El-Sharkawy, 2016). *Amaranthus* species showed large genetic variation in structural, biochemical, and physiological traits associated with photosynthesis and resource use efficiency; specific leaf weights (SLW) ranged from 20–34.2 g m^-2^, chlorophyll (Chl) 0.28–0.51 g m^-2^, nitrogen (N) 53.2–114.1 mmol m^-2^, P_n_ 19.7–40.5 μmol m^-2^ s^-1^, G_s_ 165.7–245.6 mmol m^-2^ s^-1^, photosynthetic nitrogen use efficiency (PNUE) 260–458 μmol m^-1^ Ns^-1^, and WUEp 5.6–10.4 mmol mol^-1^ (El-Sharkawy, 2016). P_n_ was positively correlated with G_s_, N and Chl of leaves, weakly correlated with SLW, and no correlation with leaf structural traits (Tsutsumi et al., 2017). Thus, *Amaranthus* species have characteristic physiological, anatomical, and biochemical traits, including high P_n_ efficiency, which are similar to C_4_ crops. Knowledge gained from *Amaranthus* may assist crop genetic enhancers to rapidly develop food, feed, and bioenergy crops with improved productivity in drylands.

To date, a set of 128 C_4_ specific genes, which are expressed in bundle sheath or mesophyll cells, are known (Ding et al., 2015). About 63% of these genes showed differential expression patterns between C_4_ [maize, sorghum, green foxtail millet (*Setaria italica* (L.) P. Beauvois)] and C_3_ (rice) species, while a smaller proportion had either novel (18%) or elevated (20%) expression patterns in C_4_ plants, which may have been involved in the evolutionary transition from C_3_ to C_4_ photosynthesis. Their differential gene expression is a novel genomic resource to establish C_4_ pathways in C_3_ crops.

Wheat is a C_3_ crop. Rangan et al. (2016a,b) discovered a C_4_ pathway restricted to wheat grain and identified six genes (*PPc, aat, mdh, me2, gpt, ppd*), specific for C_4_ photosynthesis and only expressed in the photosynthetic pericarp of the caryopsis of wheat exhibiting dimorphism related to the mesophyll- and bundle sheat cells of the chloroplasts in the leaf of C_4_ plants. Hence, it is feasible to genetically engineer C_4_ photosynthesis in wheat due to the available expression of the whole pathway in its grain, though some researchers are skeptical of this approach.

Maize is a classical C_4_ plant. The foliar leaf blade is a Kranz (C_4_) type, while the husk leaf sheath is a non-kranz (C_3_) type (Wang et al., 2013). Sixty-four of the 124 C_4_ specific genes showed differential expression between the foliar leaf blade and husk leaf sheath, of which 57 were highly expressed in the foliar leaf blade but not the leaf primordia, suggesting their involvement mainly in C_4_ photosynthesis in leaves (Ding et al., 2015). Further research on gene expression in husk leaf sheath tissues may unlock candidate genes associated with C_3_-type photosynthesis and the subsequent comparison and cross-talk between candidate genes (C_4_ and C_3_ photosynthetic pathways in maize) may unravel a cascade of genes and networks to guide further improvements in photosynthesis and productivity.

The introduction of Krantz anatomy into C_3_ leaves is a major challenge. The genes that control Krantz anatomy are unknown. Screening large-scale mutagenized populations identified variants with altered leaf anatomical structure, e.g., maize mutants with bundle sheath and mesophyll sheath specific pathways, sorghum mutants with variation in vein spacing, or rice mutants with closure vein spacing (Hall et al., 1998; Covshoff et al., 2008; Kajala et al., 2011; Rizal et al., 2015). More importantly, *Scarecrow* and *Shr1* which regulate structural differentiation of the root endodermis also contribute to the development of Kranz anatomy in maize (Slewinski et al., 2012, 2014). Thus, the discovery of such structural variation in leaf and *Scarecrow* and *Shr1* genes may provide an opportunity to introduce Krantz anatomy into C_3_ leaves.

Research to transform rice with C_4_ photosynthesis began in the last decade (Covshoff and Hibberd, 2012; von Caemmerer et al., 2012; Babege and Magule, 2017). While researchers are getting closer to unraveling the complex C_4_ photosynthesis, the pathways for such transformation will not be easy and may take another decade before this innovation can be used to introduce C_4_ photosynthesis into C_3_ crops.

Sourcing diverse germplasm pools has unraveled natural genetic variation for photosynthetic traits, both in cereals and legumes. A few QTLs and candidate genes associated with enhanced PE are known in chickpea, rice, and wheat. Significant knowledge on Krantz anatomy and key genes (and their differential expression between C_3_ and C_4_ plants) and pathways involved in the evolutionary transition from C_3_ to C_4_ photosynthesis have been unlocked. The discovery of a C_4_ pathway in wheat grain (or C_3_ pathway in maize husk) may further unravel a cascade of genes and networks to improve photosynthesis and productivity. The introduction of Krantz anatomy into C_3_ leaves is a major challenge. However, the discovery of genetic variants with altered leaf anatomical structure or genes (*Scarecrow* and *Shr1*) regulating structural differences in the root epidermis may provide opportunities for introducing Krantz anatomy into C_3_ leaves.

## Adoption, Impact, Risk Assessment, and Enabling Policy Environments

Genetically enhanced seed-embedded technology is an important innovation leading to gains in crop productivity. Both crossbreeding and biotechnology have been used to enhance tolerance to drought and heat stress in food crops. Bred-cultivars may substantially reduce projected yield losses under climate change in the tropics and subtropics (Challinor et al., 2014) while improving food self-sufficiency of smallholders and increasing their incomes without the need to cultivate extra land. Changing crop cultivars will continue to be among the first lines of defense for improving productivity and resilience in farming systems (Thornton et al., 2017).

Drought tolerant maize (DTM) cultivars developed by crossbreeding have been released in sub-Saharan Africa, while in South Asia, efforts are underway to scale up cultivation of heat stress tolerant maize hybrids. Likewise, a genetically modified drought-tolerant maize-derived hybrid, DroughtGuard (MON 87460), has been approved for cultivation in the United States (Marshall, 2014). DTM hybrids may bring benefits ranging from US$362 to 590 million for consumers and producers and could reduce poverty by 5% within 13 countries in sub-Saharan Africa (Kostandini et al., 2013). Wossen et al. (2017) showed that the adoption of DTM cultivars in rural Nigeria increased grain yields by 13.3%, and reduced grain yield variance and risk by 53 and 81% among adopters, respectively. Furthermore, productivity gains and risk reduction due to adoption reduced the incidence of poverty by 12.9% and increased the probability of food security among adopters by 83.3%. In rural Zimbabwe, adoption of DTM hybrids significantly enhanced maize productivity, allowing farmers to set aside enough produce for personal household consumption and excess for sale (Makate et al., 2017).

Adoption rates of improved seeds, regardless of their origin, in areas where those seeds are available can be as high as 85% among smallholder farmers, if awareness is high (Kyazze and Kristjanson, 2011). Many situations exist, however, where few farmers have access to improved seeds in the developing world. In sub-Saharan Africa, for example, 68–97% of seed grown by smallholder farmers comes from informal sources or local markets (McGuire and Sperling, 2015). Seed availability thus constitutes a primary barrier to the adoption of bred cultivars (Fisher et al., 2015; Lunduka et al., 2017).

DTM hybrid cultivars with big cob size, good tip cover, resistance to lodging, and good grain quality are the most preferred types in Eastern and Southern Africa (Setimala et al., 2017). Female farmers have much lower adoption of DTM, mainly due to differences in resource access, notably land, agricultural information, and credit. Male farmer age or economic status (poor or rich) has no significant influence on adoption of DTM. Young but poor female household heads are least likely to adopt DTM, while wives strongly influence the adoption of DTM on plots controlled by their husbands (Fisher and Carr, 2015). Are the farmers willing to invest more to purchase DTM seeds? Kassie et al. (2017) noted that farmers in rural Zimbabwe are willing to pay three to seven times higher premiums for drought-tolerant hybrids giving an additional metric ton of yield per acre and possessing bigger cob size, covered cob tip, and larger grains.

The environmental risks associated with the cultivation of MON 87460 are no different from the risks associated with crossbred maize (Sammons et al., 2014). Are GM crops safe for human consumption? No major differences on compositional profile were noted between GM and their counterpart, non-GM crops (Venkatesh et al., 2015; Herman et al., 2017); minor differences, however, typically reflect changes associated with crossbreeding practices (Venkatesh et al., 2015; Herman et al., 2017). Thus, GM crops are as safe and nutritious as currently consumed food crops. Robust and science-based criteria should, however, be in place to assess any biosafety issues associated with the use of such transgenic crops.

Improved understanding of how policy environments can support the widespread adoption of bred cultivars is greatly needed. There is only limited quantitative information available to guide national investment and policy choices (Vermeulen et al., 2016). Relatively small investments in cultivar development and seed multiplication now can save substantial costs later (Cacho et al., 2016), while the cost may be enormous if investment is delayed in maize breeding and seed systems (Challinor et al., 2016). It seems considerable work remains to be done with respect to the kinds of enabling policies and informational environment needed in different contexts, if adoption of bred cultivars by smallholder farmers at the scales required is to occur in the coming decades.

## Conclusion and Recommendations

Today’s agriculture is faced with increasing intensity of drought and heat stress worldwide. Greenhouse gases are the major source of global warming. The positive impact of eCO_2_ on crop productivity may be nullified or even overwhelmed by increased drought and heat stress. Developing crops that are tolerant to stress and responsive to eCO_2_ is a considerable challenge. While research on drought has long been the focus, genetic and physiological research on plant responses to eCO_2_ and heat stress has gained momentum. Tolerance to drought and heat stress is under distinct genetic control. Accessions and traits responsive to eCO_2_ (increased photosynthesis) or stress tolerance have been found in germplasm collections. The knowledge gained about the physiological and molecular bases of stress tolerance has facilitated greater access and intake of stress-tolerant germplasm by cross breeding. Advances in high-throughput genotyping and precision-based phenotyping platforms as well as the parallel development of bioinformatic resources and tools to handle large datasets are enabling crop genetic enhancers to accelerate introgression of candidate genes associated with stress tolerance into improved genetic backgrounds. Many breeding programs are routinely using these resources, which increase the speed and efficiency of selection. A few drought or heat stress-tolerant cultivars of cereals and legumes, developed by crossbreeding, are becoming popular among farmers in stress-prone environments. How are stress-tolerant crops making an impact on food and nutritional security across the globe? DTM cultivars out-yielded commercial controls on farmers’ fields, with significant gains under stress, showing the huge potential for uptake of DTM seed in Africa. However, barriers to adoption exist, such as lack of seed availability, inadequate information, resource crunch, high seed cost, and consumer preference, that must be overcome for greater uptake of seed-embedded technology to enhance food and nutritional security in the developing world.

Today, agricultural production worldwide is affected by climate change. Landraces and crop wild relatives are unique genetic resources to meet current and new challenges for farming in stressful environments. However, plant breeders are reluctant to use these unique genetic resources in breeding largely due to fear of loss of co-adapted gene complexes, linkage drag, and the protracted time needed to develop cultivars. Considerable knowledge gap also exists about crop response to eCO_2_, for example, the variance in results between controlled environment and FACE experiments, discovering plant traits most responsive to eCO_2_, or selection criterion to be adapted in breeding for the development of crop cultivars responsive to eCO_2_. Future research should focus on (i) capturing and harnessing variation in landraces and crop wild relatives, (ii) cost-effective precision-based high-throughput phenotyping platforms amenable to small-scale breeding programs, (iii) use of image-based traits (i-traits) to measure response to stress, (iv) traits and methodology to access response to selection under eCO_2_ conditions, (v) the molecular basis of plant response to selection under eCO_2_ conditions, (vi) traits, genetics, and methods to select for enhanced photosynthesis, (vii) genes and networks to unravel crosstalk involving drought × heat, genotype × eCO_2_, drought × heat × eCO_2_, and genotype × drought × heat × eCO_2_, (viii) haplotype-specific associations and protein–protein interactions to delineate natural alleles and superior haplotypes, and (ix) phenotyping and sequencing wild and weedy relative genomes of crop species to identify and relate differences with sequence variation. A focus on these issues can be expected to increase breeding efficiency to develop crops responsive to eCO_2_, stress tolerance and productivity.

## Author Contributions

All co-authors participated in planning, outlining, and writing sections of the manuscript under the lead of SD, while RO took care of the overall editing of various drafts of the manuscript after interacting with all co-authors.

## Conflict of Interest Statement

The authors declare that the research was conducted in the absence of any commercial or financial relationships that could be construed as a potential conflict of interest. The reviewer SW and the handling Editor declared their shared affiliation.
